# Corilagin Attenuates Aerosol Bleomycin-Induced Experimental Lung Injury

**DOI:** 10.3390/ijms15069762

**Published:** 2014-05-30

**Authors:** Zheng Wang, Qiong-Ya Guo, Xiao-Ju Zhang, Xiao Li, Wen-Ting Li, Xi-Tao Ma, Li-Jun Ma

**Affiliations:** 1 Department of Respiratory and Critical Medicine, the People’s Hospital of Zhengzhou University, Zhengzhou 450003, China; E-Mails: santawang99@163.com (Z.W.); lesslazy_lx@163.com (X.L.); maxitao@163.com (X.-T.M.); malijun0401@163.com (L.-J.M.); 2 Department of Gastroenterology, the People’s Hospital of Zhengzhou University, Zhengzhou 450003, China; E-Mail: santawang@outlook.com; 3 Department of Infectious Disease, Anhui Provincial Hospital, Hefei 230001, China; E-Mail: wtl9911002@163.com

**Keywords:** pulmonary fibrosis, corilagin, NF-κB, apoptosis, TGF-β1

## Abstract

Idiopathic pulmonary fibrosis (IPF) is a progressing lethal disease with few clinically effective therapies. Corilagin is a tannin derivative which shows anti-inflammatory and antifibrotics properties and is potentiated in treating IPF. Here, we investigated the effect of corilagin on lung injury following bleomycin exposure in an animal model of pulmonary fibrosis. Corilagin abrogated bleomycin-induced lung fibrosis as assessed by H&E; Masson’s trichrome staining and lung hydroxyproline content in lung tissue. Corilagin reduced the number of apoptotic lung cells and prevented lung epithelial cells from membrane breakdown, effluence of lamellar bodies and thickening of the respiratory membrane. Bleomycin exposure induced expression of MDA, IKKα, phosphorylated IKKα (p-IKKα), NF-κB P65, TNF-α and IL-1β, and reduced I-κB expression in mice lung tissue or in BALF. These changes were reversed by high-dose corilagin (100 mg/kg i.p) more dramatically than by low dose (10 mg/kg i.p). Last, corilagin inhibits TGF-β1 production and α-SMA expression in lung tissue samples. Taken together, these findings confirmed that corilagin attenuates bleomycin-induced epithelial injury and fibrosis via inactivation of oxidative stress, proinflammatory cytokine release and NF-κB and TGF-β1 signaling. Corilagin may serve as a promising therapeutic agent for pulmonary fibrosis.

## 1. Introduction

Idiopathic pulmonary fibrosis (IPF), featured by chronic exacerbating dyspnea and respiratory failure, affects 2–29 cases per 100,000 individuals [1,2]. The mean survival time of IPF after an initial diagnosis, ranging from 3.2 to 5 years, has remained unchanged in recent years despite vast improvement of novel drugs [3]. This dilemma is largely attributed to the difficulty in early identification and, more importantly, unsettlement of the pathogenetic events underlying IPF [4].

A collection of conditions is responsible for secondary pulmonary fibrosis, including acute lung injury, cigarette smoking, and environmental and drug exposure. The pathology of pulmonary fibrosis and IPF is characterized by exuberant apoptosis of lung epithelial cells and consequential fibrotic remodeling, which leads to irreversible distortion of the lung architecture and eventually honeycombing [5,6]. To date, there are no animal models that exactly mimic the pathological process of human pulmonary fibrosis, but bleomycin (BLM)-induced lung injury model is widely accepted. Either intravenous, intraperitoneal, intranasal, intratracheal or aerosolized administration of bleomycin induces lung epithelial injury and fibrosis [7,8]. Bleomycin reduces molecular oxygen to superoxide and hydroxyl radicals which cause DNA strand cleavage or breakdown [9]. Nuclear factor-κB (NF-κB) signaling is thereby provoked as a consequence of epithelial injury. Activation of NF-κB in turn promotes the release of proinflammatory cytokines such as IL-1, TNF-α, MIP-1, facilitating the chemotaxis of inflammatory cells such as circulating fibroblasts and bone-marrow mesenchymal progenitor cells into the lung [10]. NF-κB also activates TGF-β1 expression, the key mediator of pulmonary fibrosis [11,12]. TGF-β1 overexpression induces epithelial-mesenchymal transition (EMT), generates epithelia-derived fibroblasts, activates fibroblasts and fibroblast-like cells to synthesize excessive collagen and eventually induces pulmonary fibrosis [13].

Since the key steps of bleomycin-induced pulmonary fibrosis contain oxidative stress, NF-κB and TGF-β1 activation, agents directly targeting these steps are promising for reversal of this process. The efficacy of TGF-β1 inhibitor pirfenidone, free radical scavengers such as superoxide dismutase (SOD) and *N*-acetylcysteine, and NF-κB inhibitor SP100030 have been verified in experimental pulmonary fibrosis [14,15,16,17]. However, the anti-inflammatory and anti-fibrotic efficacy of these agents in animal models is much less prominent than those in preclinical tests, which suggests the complexity of the ongoing profibrotic process [4,18].

**Figure 1:**
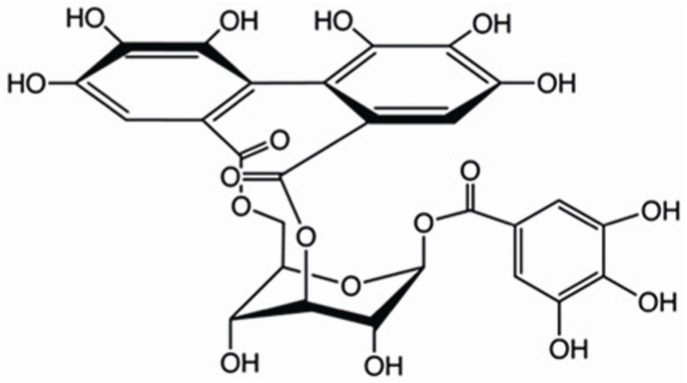
The chemical structure of corilagin (C_27_H_22_O_18_). Its molecular weight is 634.45.

Corilagin (β-d-glucopyranose, cyclic3,6-[(1R)-4,4',5,5',6,6'-hexahydroxy[1,1'-biphenyl]-2,2'-dicarboxylate]1-(3,4,5-trihydroxybenzoate) is a member of the tannin family which has been discovered in a number of medicinal plants such as *Phyllanthus amarusare* and *Geranium carolinianum* [19,20,21]. The molecular formula of corilagin is C_27_H_22_O_18_, and its molecular weight is 634.45 (Figure 1). Corilagin has shown an extensive pharmacological spectrum, including antihypertensive, antiatherogenic, antitumor and thrombolytic effects, and has potential synergic effect with beta-lactams against methicillin-resistant *Staphylococcus aureus* [19,21,22]. Corilagin inhibits NF-κB signaling and the production of proinflammatory cytokines (e.g., IL-1β and TNF-α), and is able to eliminate oxidative radicals [23,24,25,26]. The anti-inflammatory properties qualify corilagin as exempt from herpes simplex virus-1-induced microglial cell activation and cerebral damage [25], as well as to alleviate schistosomiasis liver fibrosis [27]. Attenuation of free radicals and NF-κB signaling may be part of the mechanism of alleviating liver fibrosis by corilagin, but whether corilagin inhibits TGF-β1/Smad3 signaling and epithelial-mesenchymal transition has not been ascertained [27].

Based on these data, we hypothesized that corilagin might prevent lung epithelial cells from bleomycin-induced damage via eradiation of free radicals and inhibition of NF-κB signaling. The present study was to compare the impacts of corilagin in different dosage on epithelial injury in a mice model of aerosol bleomycin-induced pulmonary fibrosis. We also investigated whether corilagin inhibits TGF-β1 signaling and collagen synthesis in this model. The findings would shed light on the effects of corilagin on bleomycin-induced lung epithelial damage and fibrosis, and unlock the possible mechanisms.

## 2. Results and Discussion

### 2.1. Macroscopic Observations

**Figure 2:**
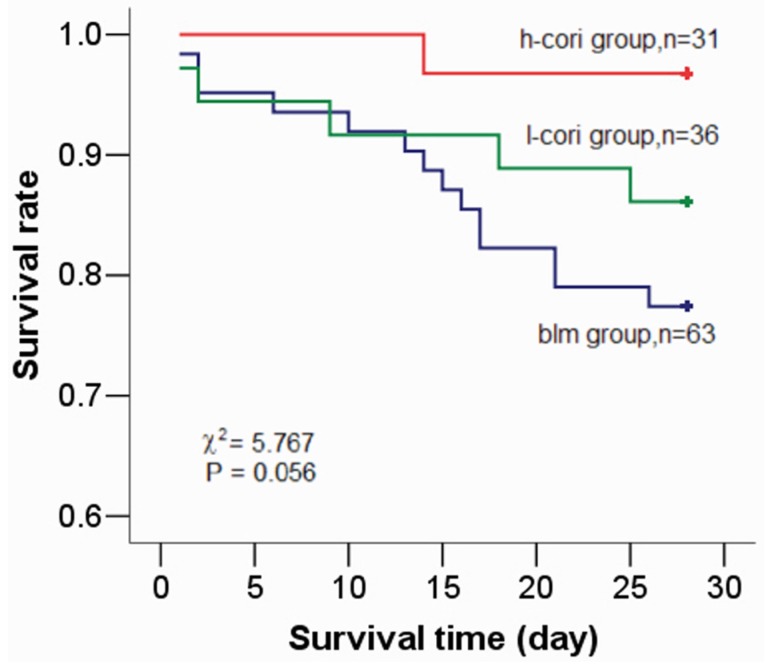
Kaplan-Meier survival curve of the mice after bleomycin treatment. There was no significant difference among survival in bleomycin exposure group (blm), bleomycin exposure and 10 mg/kg corilagin group (l-cori) and bleomycin exposure and 100 mg/kg corilagin group (h-cori) (χ^2^ = 5.767, *p* = 0.056).

One hundred and forty-three mice were included in the preliminary study and in the formal study. The overall survival rate was 86.0% (122/143). Survival rates in the control group (ctrl), the bleomycin exposure group (blm) group, the bleomycin exposure+corilagin 10 mg/kg group (l-cori) and the bleomycin exposure+corilagin 100 mg/kg group (h-cori) were 100% (14/14), 77.4% (48/62), 86.1% (31/36) and 96.8% (30/31), respectively (χ^2^ = 9.07, *p* = 0.024). The survival curve showed that there were no significance among survival in the blm group, the l-cori group and h-group (χ^2^ = 5.767, *p* = 0.056) (Figure 2). There were one death in the blm+cori d15–28 group (bleomycin exposure+corilagin d15–28), three in the blm group (bleomycin exposure), and none in the blm+cori d1-14 (bleomycin exposure+corilagin d1–14 group). Bleomycin-treated animals had anorexia more often than in the control group. The mean body weight of the ctrl group, the blm group, the l-cori group, and the h-cori group on day 29 was (25.3 ± 2.6), (18.6 ± 2.7), (21.3 ± 3.2) and (24.4 ± 3.0) g (*p* = 0.033), as shown in Table 1.

**Table 1:** The hydroxyproline content, fibrosis score and BALF cell count in four groups (mean ± SD, *n =* 5 or 7 for each situation).

Characteristics	Group				F Value	*p* Value
	ctrl	blm	l-cori	h-cori		
Survival rate (%)	100(14/14)	77.4(48/62)	86.1(31/36)	96.8(30/31)	9.07 ^†^	0.024
Body weight (g)						
d0	19.0 ± 0.7	19.2 ± 0.8	18.9 ± 0.7	19.0 ± 0.6	0.244	0.78
d28	25.3 ± 2.6	18.6 ± 2.7	21.3 ± 3.2 *^#^	24.4 ± 3.0 ^#^	1.764	0.033
Ashcroft score						
d7	0.4 ± 0.1	0.7 ± 0.2	1.0 ± 0.4 *	0.9 ± 0.3 *	6.533	0.002
d15	0.8 ± 0.2	3.4 ± 0.7 *	2.5 ± 0.6 *	1.9 ± 0.7 *^#^	24.145	<0.001
d28	1.4 ± 0.2	5.1 ± 1.0 *	3.9 ± 0.8 *	2.8 ± 0.8 *^#^	30.076	<0.001
Hydroxyproline (mg/g)						
d7	1.63 ± 0.11	1.85 ± 0.30 *	1.83 ± 0.33	1.91 ± 0.24 *	2.322	0.011
d15	1.82 ± 0.27	3.76 ± 0.57 *	2.88 ± 0.53 *^#^	2.44 ± 0.41 *^#^	18.675	<0.001
d28	2.20 ± 0.13	7.34 ± 0.92 *^#^	5.10 ± 0.62 *^#^	3.64 ± 0.58 *^#^	85.053	<0.001
BALF cell count (10^4^/mL)						
d7	8.7 ± 1.6	22.3 ± 3.4	16.7 ± 1.2	13.4 ± 1.8	17.456 ^§^	<0.001
d15	9.0 ± 1.0	19.9 ± 2.0 *	14.0 ± 2.0 *^#^	12.7 ± 1.2 *^#^	38.19	<0.001
d28	8.6 ± 1.4	17.9 ± 2.5 *	12.4 ± 2.7 ^#^	13.0 ± 2.5 ^#^	13.244	<0.001
BALF differential cell proportion						
Macrophages						
d7	97.9 ± 0.5	57.7 ± 6.3 *	63.2 ± 5.4 *	69.5 ± 11.7 *^#^	44.167	<0.001
d15	96.3 ± 0.4	56.1 ± 7.7 *	57.8 ± 8.4 *^#^	65.3 ± 7.7 *^#^	35.945	<0.001
d28	96.1 ± 0.8	46.6 ± 8.2 *^#^	73.7 ± 8.4 *^#^	67.7 ± 7.5 *^#^	42.586	<0.001
Neutrophils						
d7	0.8 ± 0.2	10.0 ± 1.4 *	14.2 ± 2.3 *	15.2 ± 2.5 *	64.645	<0.001
d15	2.4 ± 0.2	12.4 ± 2.3 *	15.8 ± 3.5 *	16.5 ± 5.2 *	18.630	<0.001
d28	2.5 ± 0.1	7.7 ± 1.8 *	11.3 ± 2.3 *	21.8 ± 5.2 *^#^	38.462	<0.001
Lymphocytes						
d7	1.2 ± 0.2	33.4 ± 6.5 *	22.3 ± 4.8 *^#^	15.3 ± 3.6 *^#^	47.488	<0.001
d15	1.1 ± 0.2	31.2 ± 5.2	24.7 ± 4.6 *	17.3 ± 5.5 *^#^	42.800	<0.001
d28	1.4 ± 0.3	24.9 ± 4.3 *	15.4 ± 5.2 *^#^	10.5 ± 4.3 *^#^	29.966	<0.001

^†^: χ^2^ value for Chi-square test; ^§^: χ^2^ value for Kruskall-Wallis rank sum test; *: *p* < 0.05 compared with the ctrl group; ^#^: *p* < 0.05 compared with the blm group.

### 2.2. Corilagin Ameliorates Bleomycin-Induced Pulmonary Fibrosis

**Figure 3:**
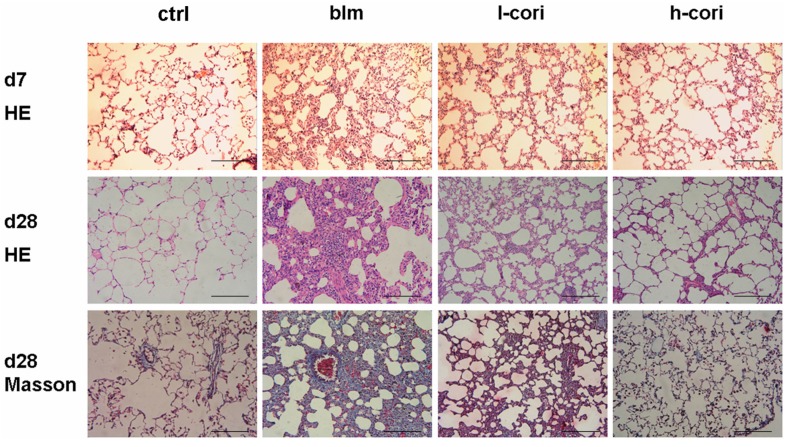
Representative lung pathology images on days 7 and 28 in each group. Dramatic alveolar and massive septal collagen deposition were manifested in the blm group, followed by l-cori group, while the lung architecture remained intact in the h-cori group. Collagen was stained blue with Masson’s trichrome. H&E and Masson’s trichrome, scale bars represent 200 µm.

**Figure 4:**
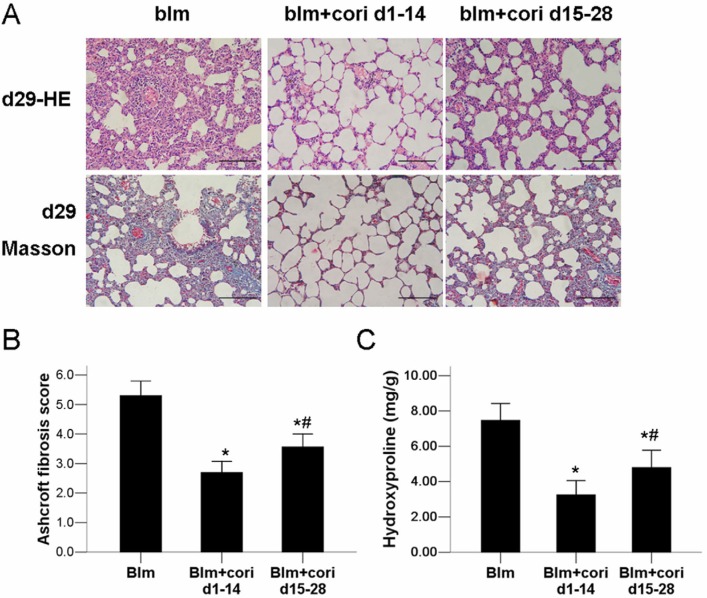
The protective effect of in corilagin on bleomycin-induced lung pathology is time-dependent. (**A**) Representative lung pathology at day 29 in mice without (blm group) or with i.p. injection of corilagin (100 mg/kg) from days 1–14 (blm+cori d1–14 group) or days 15–28 (blm+cori d15–28 group); (**B**) Ashcroft fibrosis score in each group; (**C**) Hydroxyproline content in each group. H&E and Masson’s trichrome, scale bars represent 200 µm. *: *p* < 0.05 compared with the blm group; #: *p* < 0.05 compared with the blm+cori d1–14 group.

Lung pathology was assessed by H&E and Masson’s trichrome. Lung sections of the blm group and the l-cori group obtained at day 7 showed inflammatory cell infiltration and alveolitis (Figure 3). Diffuse fibrosis with cellular infiltration, alveolar wall thickening and destruction of the alveolar structure were recognized in the blm group at day 28 (Figure 3). In the l-cori group, the degree of septa thickening, alveolar destruction and collagen accumulation (blue-stained area on Masson’s trichrome) was weakened compared with those of the blm group (Figure 3). Control tissue showed normal findings over time. Figure 3 shows that i.p. injection of corilagin from days 1–14 and 15–28 attenuated bleomycin-induced pulmonary fibrosis and hydroxyproline production. As shown via one-way ANOVA analysis, the fibrosis score and lung hydroxyproline content were higher in blm+cori d15–28 group than in blm+cori d1–14 group, but were both lower than those in the blm group (Figure 4). As shown in Table 1, The Aschroft’s fibrosis scores assessed at day 28 in the ctrl group, the blm group, l-cori group and h-cori group were 1.4 ± 0.2, 5.1 ± 1.0, 3.9 ± 0.8 and 2.8 ± 0.8, respectively (*p* < 0.001, Kruskall-Wallis rank sum tests). High-dose corilagin (100 mg/kg) decreases the Aschroft’s fibrosis score more significantly than low-dose corilagin (10 mg/kg). The content of hydroxyproline in lung tissue (mg/g) at 28th day in the blm group was 7.34 ± 0.92, increased significantly compared with the ctrl group (2.20 ± 0.13), but was slightly reduced in the l-cori group (5.10 ± 0.62) and more in the h-cori group (3.64 ± 0.58) (Table 1). Also to note is that corilagin administration alters the BALF cell count and differential cell proportion (Table 1).

### 2.3. Corilagin Alleviates Bleomycin-Induced Lung Epithelial Injury

**Figure 5:**
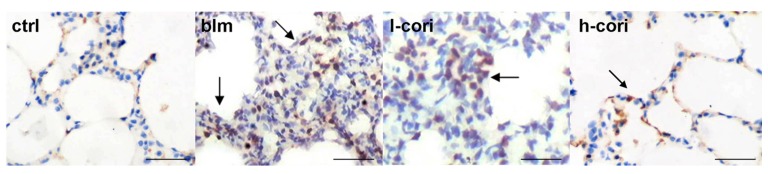
Corilagin alleviated bleomycin-induced lung epithelial apoptosis. Apoptotic cell numbers per each 200 times-magnified field were manifested by terminal deoxynucleotidyl transferase (TdT)-mediated dUTP nick end labeling (TUNEL). Lung tissue samples were obtained 24 h after bleomycin exposure. Apoptotic cell nuclei are brown-stained by TUNEL and marked with arrows, originally magnification, scale bars represent 100 µm. Apoptotic cell numbers per each field were 1.6 ± 0.8, 34.6 ± 8.2, 26.3 ± 9.6 and 16.0 ± 7.3 in the ctrl group, the blm group, l-cori group and h-cori group (*p* < 0.01).

**Figure 6:**
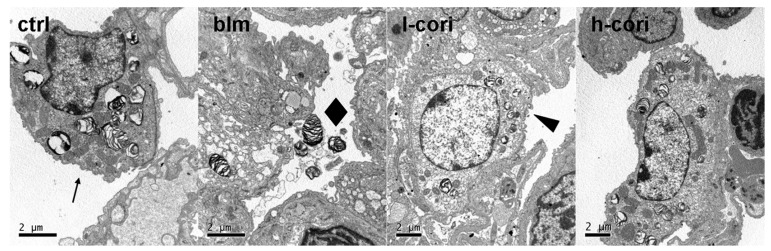
The ultrastructure of mice lungs observed through a transmission electron microscopy 24 h after bleomycin exposure. Arrow (←): lamellar bodies; diamond (◆): effluence of lamellar bodies; arrowhead (◄): swell of nucleus and plasma of alveolar epithelia.

Corilagin alleviated epithelial apoptosis in a dose-dependent manner after bleomycin inhalation (Figure 5). Apoptotic cell numbers per each 200 times-magnified field were 1.6 ± 0.8, 34.6 ± 8.2, 26.3 ± 9.6 and 16.0 ± 7.3 in the ctrl group, the blm group, l-cori group and h-cori group, respectively (*p* < 0.01, Kruskall-Wallis rank sum tests). Figure 6 reflects the lung ultrastructure of BALB/c mice 24 h later to interventions. The ctrl group showed a normal ultrastructure of type II pneumocyte and respiratory membrane; the blm group showed breakdown of cell membrane and subcellular structures, effluence of lamellar bodies and thickening of respiratory membrane in the contiguous area, which suggests alveolar epithelial injury; the l-cori group showed swelling of organelles and plasma of alveolar epithelia; the AT II cells retain their shape in the h-cori group which is similar to the ctrl group.

### 2.4. Corilagin Reduces Bleomycin-Induced Oxidative Responses and NF-κB Activation

**Figure 7:**
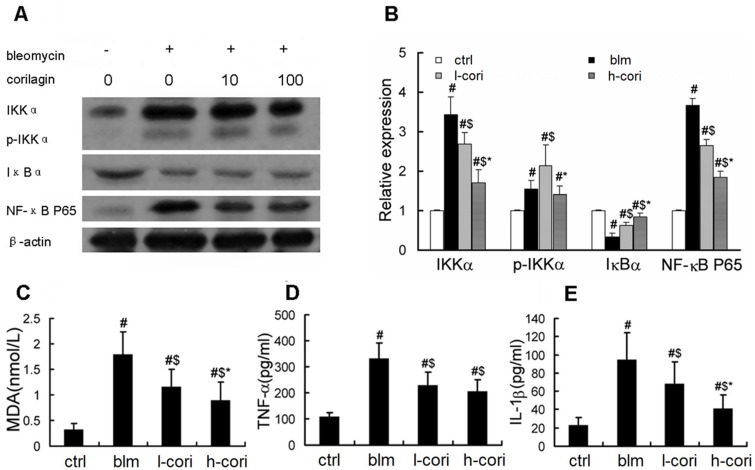
Corilagin reduced bleomycin-induced lipid peroxidation and NF-κB activation. (**A**) Western blot analysis of IKKα, phosphorylated IKKα (p-IKKα), I-κBα and NF-κB P65; (**B**) Fold increases of NF-κB signaling proteins. Densometry was normalized by β-actin. Experiments were repeated three times; (**C**) BALF content of malondialdehyde (MDA); (**D**) BALF level of TNF-α; (**E**) BALF level of IL-1β. #: *p* < 0.05 compared with the ctrlgroup; $: *p* < 0.05 compared with the blm group; *: *p* < 0.05 compared with the l-cori group; *N =* 7–10 for each group.

Oxidative responses, NF-κB activation and proinflammatory cytokine production 7 days after bleomycin exposure was inhibited by corilagin at a dose-dependent manner. Bleomycin induced expression of IKKα, phosphorylated IKKα (p-IKKα), NF-κB P65, TNF-α and IL-1β, and reduced I-κBα expression in mice lung tissue or in BALF. This change was reversed in the h-cori group more dramatically than in the l-cori group (Figure 7A,B,D,E). The average BALF level of MDA, a lipid peroxidation marker, was (0.32 ± 0.13) nmol/L at baseline and increased to (1.79 ± 0.45) nmol/L after bleomycin inhalation, and was slightly reduced in the l-cori group ((1.17 ± 0.33) nmol/L) and more in the h-cori group ((0.90 ± 0.35) nmol/L) (*p* = 0.021, one-way ANOVA) (Figure 7C). 

### 2.5. Corilagin Decreases TGF-β1 Production and α-SMA Expression

Corilagin significantly reduced α-SMA expression as assessed by immunohistochemistry (Figure 6A,B). As shown in Figure 6C, The TGF-β1 content in lung tissue samples (ng/mg) were 0.27 ± 0.06, 2.14 ± 0.37, 1.45 ± 0.20 and 0.93 ± 0.18 in the ctrl group, the blm group, l-cori group and h-cori group, respectively (*p* < 0.01, Kruskall-Wallis rank sum tests).

### 2.6. Discussion

This study shows that corilagin attenuated bleomycin-induced pulmonary fibrosis in mice. Corilagin alleviates epithelial damage and apoptosis, but only high-dose corilagin preserved the lung architecture, whereas low-dose corilagin moderately ameliorated lung epithelial damage and lung fibrosis. This lung-protective effect of corilagin is associated with the reduction of lipid peroxidation and inhibition of NF-κB signals, and alleviation of epithelial damage. In addition, corilagin inhibited collagen synthesis and TGFβ1 production, which may add to its anti-fibrotic activities.

Bleomycin-induced lung fibrosis is primarily mediated by reactive oxygen species (ROS). The lung harboring low levels of bleomycin hydrolase are susceptible to bleomycin exposure and subsequent excessive production of ROS. ROS activated the transcription of inflammatory genes, causing apoptosis and cell membrane injury through lipid peroxidation in lung epithelial cells [28]. Insufficient epithelial regeneration following widespread ATII cell damage resulted in the fibrotic pattern like a scar in the lung. The significance of ATII cell apoptosis is evidenced by reversal of bleomycin-induced lung fibrosis by intratracheal transplantation of alveolar type II cells [29]. In this study, i.p administration of corilagin dose-dependently reduced the level of MDA, a lipid peroxidation marker (Figure 7C). Corilagin also abrogated apoptosis, breakdown of cell membrane and subcellular structures, effluence of lamellar bodies and thickening of respiratory membrane in ATII cells 24 h after bleomycin inhalation (Figure 5 and Figure 6). These results were in line with previous studies, which showed the efficacy of ROS scavengers such as superoxide dismutase (SOD) and *N*-acetylcysteine in prevention of bleomycin-induced lung epithelial damage and fibrosis [15,16]. Bleomycin-induced lung epithelial injury is mediated by free radicals, which are produced mostly in the first 24 h after bleomycin exposure [30]. In this regard, corilagin protects ATII cell damage via scavenging ROS generated in response to bleomycin exposure in the very early stage, which may serve as its first antifibrotic mechanism.

Activation of NF-κB signaling and breakout of proinflammatory mediators are well documented in bleomycin-treated lung tissues, and inhibition of these signals is the second possible mechanism for corilagin. In this study, we identified the inhibitory effect of corilagin on production of TNF-α, IL-1β and NF-κB activation (Figure 7). TNF-α is one of the utmost cytokines reactive to proinflammatory irritants, which induces the production of other cytokines and growth factors (e.g., IP-10, MIP1α, *et al*.) capable of mediating leukocyte chemotaxis. TNF-α also activates the caspases, Bax/Bak and NF-κB apoptotic signals, which aggregates alveolar damage. Alveolar damage activates the expression of TNF-α in turn and forms the “damage-inflammation” positive feedback loop. Except for induction of inflammation, TNF-α upregulates the expression of TGF-β1, the most renowned profibrotic factor [31]. TNF-α blockade with either anti-TNF-α antibodies or TNF-α antagonists can inhibit fibrosis [32]. Thus, it is conceivable that corilagin injection results in a reduced inflammatory infiltrate in the lung following bleomycin administration and reduced TGF-β levels in this model. The case is similar with IL-1β. IL-1β, which acts synergistically with TNF-α in activation of NF-κB, is upregulated following bleomycin administration [33]. Transient overexpression of IL-1β induces lung injury and pulmonary fibrosis in the late stages of the experimental setting, while inhibition of IL-1β prevented the fibrosing reaction induced by bleomycin in mice [34]. In the current study, inhibition of TNF-α, IL-1β and NF-κB by corilagin is associated with the decrease of BALF cell numbers, particularly lymphocytes (Table 1, Figure 7). These data indicate that corilagin possibly inhibits bleomycin-induced NF-κB activation and subsequent cytokine release by T lymphocytes, resulting in the blockage of the inflammatory cascade. Moreover, bleomycin exposure induced the lung infiltration of almost all inflammatory cells, which were similar to that of previously described [6,7]. Interestingly however, corilagin reduced bleomycin-induced lung lymphocyte infiltrations while decreases the number of lung neutrophils (Table 1). We assume that corilagin alleviated early-stage lung inflammation and specifically, neutrophilic inflammation, via inhibition of TNF-α, IL-1β and NF-κB. While the proportions of neutrophils were prominently reduced by corilagin, the proportions of other cells (alveolar macrophages, lymphocytes, *etc*.) increased subsequently. At the same time, the total cell numbers in BALF were greatly reduced as a result of corilagin treatment. While in the fibrosis stage, corilagin may inactivate lymphocytes more than neutrophils. The inhibitory discrepancies may be associated with certain sorts of chemokines. More in depth study is needed, as this study did not provide direct evidence of the lymphocyte subtype involved with the anti-inflammatory property of corilagin.

**Figure 8:**
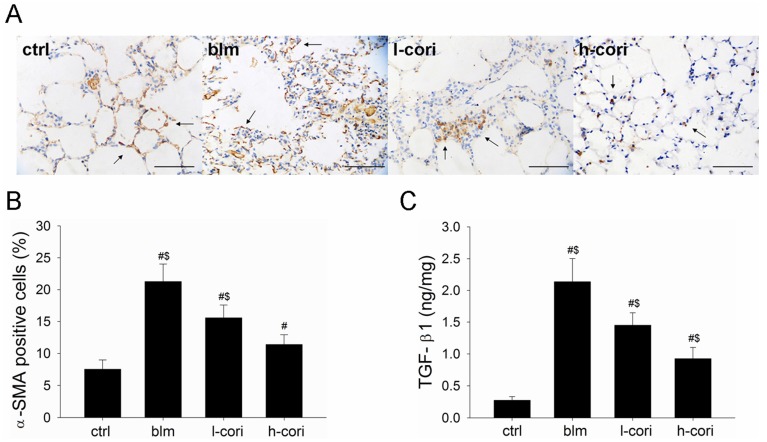
Corilagin inhibited α-SMA expression and TGF-β1 production after bleomycin-induced lung injury. (**A**) Immunohistochemical staining of α-SMA. The whole cells were brown-stained (arrow). Scale bars represent 200 µm; (**B**) Average proportions of α-SMA positive cells in 200 times-magnified fields; (**C**) Content of TGF-β1 in lung homogenate samples. #: *p* < 0.05 compared with the ctrl group; $: *p* < 0.05 compared with the blm group.

It is noteworthy that administration of corilagin from days 15–28 also attenuated bleomycin-induced pulmonary fibrosis, suggesting that corilagin might possess a direct antifibrotic role. In addition to free radical elimination and NF-κB inactivation, the direct anti-fibrotic activity of corilagin is noteworthy with TGF-β1 production and collagen synthesis, which accounts for its pharmacological effects on experimental lung fibrosis (Figure 8). As the key mediator of organ fibrosis, TGF-β1 activates fibroblasts to synthesize collagen, and promotes them to express α-SMA and become myofibroblasts [12,13,18]. TGF-β1 also induces the epithelial-to-mesenchymal transition (EMT) process in ATII cells, in which localized ATII cells undergo a shift towards mesenchymal phenotype and begin to synthesize collagen and α-SMA [13]. The transformed α-SMA positive “ATII” cells and myofibroblasts express higher levels of ECM and TGF-β1 than their α-SMA negative counterparts [35]. TGF-β1 is released by injured ATII and smooth muscle cells, as well as activated lung fibroblasts in an autocrine manner [36]. TGF-β1 inhibition by pirfenidone ameliorates bleomycin-induced fibrosis in animals as well as in human IPF [14]. Here, we provides evidences that corilagin inhibits both TGF-β1 production and α-SMA expression *in vivo* in the model of lung fibrosis (Figure 8). As shown in Figure 7, corilagin attenuates TNF-α, IL-1β and NF-κB expression, which are all potential profibrotic signals that activate TGF-β1 expression. In this regard, we propose that the inhibitory effects of corilagin on TGF-β1 production *in vivo* may stem from indirect attenuation of proinflammatory signals. However, whether corilagin directly suppresses TGF-β1 autocrine in lung fibroblasts remains to be elucidated.

As a member of the tannins family, corilagin has three 1,2,3-trihydroxybenzene groups and one glucose group in the chemical structure (Figure 1), which are hydrogen donors yielding its anti-oxidant activities. Corilagin inactivates free radicals through supply of hydrogen like other polyphenols [37]. The mechanism of corilagin in reduction of proinflammatory cytokines and NF-κB signaling is possibly associated with induction of inducible nitric oxide synthase (iNOS) [38]. It was recently shown that tannins inhibits the activity of apoptosis-promoting poly (ADP-ribose) glycohydrolase (PRAP) [39]. The anti-inflammatory properties of ginger extract has been attributed to inhibition of prostaglandin biosynthesis, cyclooxygenase (COX)-1 and COX-2, leukotriene biosynthesis by inhibiting 5-lipoxygenase, and directly attenuating the expression of inducible proinflammatory genes encoding cytokines, chemokines and inducible COX-2 [40]. Corilagin showed similar effects on reduction of proinflammatory cytokines, NF-κB signaling and oxidative stress after bleomycin exposure in the lung like other tannins. However, it is unknown whether corilagin acts in the same way as ginger extract in the biotransformation of arachidonic acid, or inhibits the activity of PRAP.

This study highlights an important move towards the cure of pulmonary fibrosis: natural botanical products which block more than one crucial step in the fibrotic process. The molecular targets of corilagin include peroxidation, type I alveolar inflammation, NF-κB signaling and TGF-β1 expression (Figure 3,Figure 4, Figure 5, Figure 6, Figure 7,and Figure 8). In a word, corilagin might have both direct antifibrotic (reducing TGF-β1 expression) and indirect antifibrotic properties (alleviating lung peroxidation, infiltration and NF-κB signaling). However, to date we are unaware which one presides. Till now, treatment options efficient in experimental lung fibrosis or human IPF include the anti-oxidants, anti-fibrinolytic agents (urokinase), cytokine inhibitors (corticosteroids), cytotoxic agents (azathioprine), antifibrotics (pifernidone), stem cell engraftments (mesenchymal stem cells or embryonic stem cells), and gene therapy targeting Wnt/β-catenin signaling or else. These are confined to mostly focusing on one or two aspects in the process of lung injury and repair. In comparison, as shown above, corilagin attenuates bleomycin-induced pulmonary fibrosis though inhibition of NF-κB, lipid peroxidation and TGF-β1 signaling in lung tissue. The wider action spectrum of corilagin than previously attempted options might make it a more promising agent for pulmonary fibrosis. However, the efficacy of corilagin in experimental fibrosis is not warranted further in human IPF, since the ongoing profibrotic process in IPF is far more complicated than that in experimental lung fibrosis. The cause of epithelial injury is clear in experimental lung fibrosis but cryptogenic in IPF. Bleomycin-induced pulmonary fibrosis could be divided into three stages—injury, inflammation and fibrosis—which cover less than 4 weeks in all [30]. In comparison, human IPF is a chronic pathological process where epithelial injury, inflammation, mal-regeneration and fibrosis co-exist. These discrepancies well account for the fact that the anti-fibrotic efficacy of these agents obtained in IPF patients does not parallel that in animal models [3,22]. Therefore, further studies on corilagin are needed to fill the gap between the bench in alleviating experimental pulmonary fibrosis and the bedside in treating IPF.

## 3. Experimental Section

### 3.1. Animals

Male BALB/c mice which initially weighed between 18.0 and 20.0 g were purchased from the Experimental Animal Center, Anhui Medical University. Mice were randomized according to their body weight to the control group (ctrl group), the bleomycin-exposure group (blm group), the bleomycin exposure and 10 mg/kg corilagin group (l-cori group), and the bleomycin exposure and 100 mg/kg corilagin group (h-cori group). The average body weight was (19.0 ± 0.7) g (con group), (19.2 ± 0.8) g (blm group), (18.9 ± 0.7) g (l-cori group), (19.0 ± 0.6) g (h-cori group), which did not differ among each group after randomization (Table 1). Animals were kept in a relatively clean environment with constant temperature and humidity, and had free access to the pellet diet and water *ad libitum*. They had been fed 1 week prior to the experiment. Animals were treated in a manner that summarized suffering. All the experimental procedures were approved by the Animal Care and Use Committee of Anhui Medical University, and were in accord with the Guide for the Care and Use of Laboratory Animals issued by the US National Institutes of Health.

In order to investigate whether the *in vivo* anti-fibrotic effect of corilagin in bleomycin-induced pulmonary fibrosis was time-dependent, an additional experiment was performed. Another group of mice were randomized to three groups: the bleomycin-exposure group (blm group), the bleomycin exposure+100 mg/kg corilagin days 1–14 group (blm+cori d1–14 group), the bleomycin exposure+100 mg/kg corilagin days 15–28 group (blm+cori d15–28 group). Mice were exposed to bleomycin at day 1, and then were injected with DMSO or DMSO-corilagin solution as indicated. Then, mice were sacrificed at day 29, and lung pathology and hydroxyproline content were studied.

### 3.2. Inhalation of Bleomycin and Administration of Corilagin

We performed aerosol bleomycin exposure following the methods by Li *et al*. with some modifications [8]. A pathogen-free inhalation plexiglass apparatus (50 × 40 × 30 cm) was used for passive bleomycin exposure. Bleomycin aerosol was produced by an ultrasonic nebulizer (Anshan Medical Electronic Apparatus Co., Anshan, Liaoning, China) and was conducted into the inhalation apparatus. The nebulizer yields a steady airflow of 40 L/min. Four micrograms of bleomycin A2 (Nippon Kayaku Co., Ltd., Tokyo, Japan) dissolved in 4 mL of sterilized saline (1 mg/mL) was aerosolized each time. Nebulization lasted for approximately 8 min. Mice were kept in the inhalation apparatus for 30 min overall. After bleomycin exposure was left off, fresh air was introduced and animals were taken away. The inhalation apparatus was sterilized with 75% ethanol after use and ultraviolet rays 30 min before use. Dosing of bleomycin and corilagin in this step was determined according to pharmacodynamic and pretest data. Corilagin (Kouting Chemical Co., Ltd., Shanghai, China; purity ≥ 99.0%) was dissolved in dimethyl sulfoxide (DMSO) at a concentration of 1 or 10 mg/mL. The volume of corilagin solution (approximately 0.2 mL) allocated intraperitoneally each time was calculated according to the body weight of each mice, yielding a dose of 10 mg/kg (low dose) or 100 mg/kg (high dose), respectively. Control mice and the bleomycin exposure group mice were intraperitoneally injected with an equivalent amount of DMSO instead. All mice underwent intraperitoneal injection once daily for 14 consecutive days. At day 1, the bleomycin exposure, lasting for 30 min as mentioned above, was performed 15 min after injection of corilagin. Bleomycin-induced lung epithelial injury is mediated by free radicals, which are produced mostly in the first 24 h after bleomycin exposure [30]. So, we checked the content of free radicals and apoptotic status of epithelial cells 24 h after exposure. In the supplemented experiment, all mice were exposed to aerosol bleomycin at day 1. Intraperitoneal injection was executed in the blm group with DMSO (0.2 mL for days 1–28), in the blm+cori d1–14 group with corilagin (100 mg/kg, dissolved in 0.2 mL DMSO) for consecutive days 1–14 and DMSO for days 15–28, and in the blm+cori d15–28 group with corilagin for consecutive days 15–28 and DMSO for days 1–14, respectively.

### 3.3. Bronchoalveolar Lavage (BAL) and Sample Collection

On days 2, 7, 14 and 29, mice were anesthetized by injection of sodium pentobarbital (300 mg/kg intraperitoneally). Immediately thereafter, a midline neck incision was made, and the trachea was cannulated. The left lung was lavaged using 2.4 mL cooled PBS, 0.8 mL each time for three times. The lavage (BALF) gained by three times of BAL was collected together. BALF cells were stained by Wright-Giemsa and counted on a hemocytometer. To retrieve a statistically reliable differential estimation of BALF cells, 400 cells were differentiated and counted each time. The BALF samples were allocated into two parts: the first part was used for MDA detection, the other part was then frozen at −70 °C till analysis. Right lung was harvested and frozen at −70 °C for immunoblot assay and measurement of hydroxyproline (HYP) content. A small piece of the right lung (approximately 2 × 2 × 2 mm) was fixed in 3% glutaraldehyde and sent for ultra-morphological examination within 2 h. The remaining right lungs were fixed in 4% paraformaldehyde before histological analysis.

### 3.4. Measurement of Oxidative Stress

We measured the level of malondialdehyde (MDA) in BALF to quantify oxidative stress. All the procedures closely followed the instructions of a commercial kit (Beyotime Institute of Biotechnology, Haimen, Jiangsu, China). BALF was incubated with 0.1% thiobarbituric acid for 15 min at 100 °C. The absorbance of the solution was measured at 532 nm and standardized for protein content. The standard absorbance curve was drawn and the content of MDA was calculated.

### 3.5. Hydroxyproline Assay

The measurement of hydroxyproline in lung tissue was performed using a hydroxyproline detecting kit (Jiancheng Bioengineering Institute, Nanjing, Jiangsu, China) by chloramine hydrolysis method, as previously described [41].

### 3.6. Enzyme-Linked Immunosorbent Assay (ELISA)

The levels of TNF-α, IL-1β and TGF-β1 in BALF samples were measured by closely following the instructions of commercially available ELISA kits (R& D Systems Co., Ltd., Minneapolis, MN, USA). Standard absorbance curve and equation were established, concentrations of cytokines and collagen were then calculated accordingly.

### 3.7. Lung Morphology

For microscopic observation, the lung samples were fixed by 4% paraformaldehyde for 24 h, and then prepared on lung sections and undertook standard H&E and Masson’s trichrome staining. Lung sections were observed under microscope and the grade of inflammation and fibrosis were semi-quantitatively analyzed according to the method described by Ashcroft *et al*. [42]. For transmitting electron microscopic analysis, lung samples (obtained at day 2) fixed in glutaraldehyde were re-fixed in osmic acid, dehydrated, stained using lead citrate, prepared in ultrathin sections and observed using a JEM-2100F transmitting electron microcopy (Hitachi, Hitachi, Japan).

### 3.8. Apoptosis Assay by Terminal Deoxynucleotidyl Transferase (TdT)-Mediated dUTP Nick end Labeling (TUNEL)

The TUNEL assay was carried out using a TUNEL staining kit according to the manufacturer’s instructions (Roche, Germany). Enzyme solution containing TdT and labeling solution containing biotinized dUTP were added to the TUNEL reaction solution at a dilution of 1:9. Cells with brown-stained nuclei or those containing apoptotic bodies were considered apoptotic. Apoptotic cells were counted by a blinded investigator on 200 times-magnified fields. Ten vision fields from 5 random areas were observed on each section.

### 3.9. Immunohistochemistry

Immunohistochemistry of α-SMA was performed on deparaffinated sections with a rabbit polyclonal α-SMA antibody (Abcam Ltd., Cambrigde, UK) as mentioned before [41]. Staining of α-SMA were developed using a DAB color developing kit after affiliation of SABC-conjugated secondary antibody. The operating concentration of α-SMA was 1:400.

### 3.10. Western Blot Assay

Western blot was carried out with mice lung tissue or cell culture using commercial kits as previously described [41]. Rabbit anti-mouse IKKα, phosphorylated IKKα, IκB-α, NF-κB P65 and β-actin primary antibodies were purchased from Santa Cruz Biotechnology (Santa Cruz, CA, USA). The operating concentration of primary antibodies ranged from 1:300–1:400.

### 3.11. Statistical Analysis

Data were analyzed by SPSS 15.0 statistical package (SPSS Inc., Chicago, IL, USA). Results were presented as mean ± SD. Statistical significance among the group means was assessed by one-way analysis of variance (ANOVA). Post hoc testing was performed with Bonferroni’s modification of the *t*-test. Kruskal-Wallis rank sum test was used alternatively while the data were inappropriate for ANOVA analysis (e.g., Data were not normally-distributed). The constituent ratios were compared using the χ^2^ test (Chi-square test) and the Fisher exact test. Survival data were analyzed by Kaplan-Meier analysis. Differences were considered significant at *p* < 0.05.

## 4. Conclusions

Taken together, corilagin attenuates lung injury and fibrosis as a consequence of bleomycin inhalation. This lung-protective effect is attributed to its anti-oxidative, anti-inflammatory and anti-apoptotic activities, as well as inhibition of TGF-β1 expression and ECM synthesis. Blockage of several key events in bleomycin-induced lung fibrosis renders corilagin as a promising anti-fibrotic agent for IPF. We suggest that corilagin might be potentiated in the clinical setting and warrants further study.
